# Heterologous Expression of Ilicicolin H Biosynthetic Gene Cluster and Production of a New Potent Antifungal Reagent, Ilicicolin J

**DOI:** 10.3390/molecules24122267

**Published:** 2019-06-18

**Authors:** Xiaojing Lin, Siwen Yuan, Senhua Chen, Bin Chen, Hui Xu, Lan Liu, Huixian Li, Zhizeng Gao

**Affiliations:** 1 School of Marine Sciences, Sun Yat-sen University, Guangzhou 510006, China; linxiaojing003@126.com (X.L.); swy1101@163.com (S.Y.); chensenh@mail.sysu.edu.cn (S.C.); chenbin25@mail.sysu.edu.cn (B.C.); cesllan@mail.sysu.edu.cn (L.L.); 2 Research Center of Chinese Herbal Resource Science and Engineering, Guangzhou University of Chinese Medicine, Guangzhou 510006, China; zyfxsherry@gzucm.edu.cn; 3 Southern Marine Science and Engineering Guangdong Laboratory (Zhuhai), Zhuhai 519080, China

**Keywords:** ilicicolin H, *Neonectria* sp. DH2, heterologous production, biosynthetic gene cluster

## Abstract

Ilicicolin H is a broad-spectrum antifungal agent targeting mitochondrial cytochrome bc1 reductase. Unfortunately, ilicicolin H shows reduced activities in vivo. Here, we report our effort on the identification of ilicicolin H biosynthetic gene cluster (BGC) by genomic sequencing a producing strain, *Neonectria* sp. DH2, and its heterologous production in *Aspergillus nidulans*. In addition, a shunt product with similar antifungal activities, ilicicolin J, was uncovered. This effort would provide a base for future combinatorial biosynthesis of ilicicolin H analogues. Bioinformatics analysis suggests that the backbone of ilicicolin H is assembled by a polyketide-nonribosomal peptide synthethase (IliA), and then offloaded with a tetramic acid moiety. Similar to tenellin biosynthesis, the tetramic acid is then converted to pyridone by a putative P450, IliC. The decalin portion is most possibly constructed by a *S*-adenosyl-l-methionine (SAM)-dependent Diels-Alderase (IliD).

## 1. Introduction

Nature is a great resource to produce natural products as tools to investigate biological problems, and many natural products or their derivatives have been developed into pharmaceutical agents [1]. Filamentous fungi produce many bioactive metabolites that have been used as medicine, such as penicillin, cyclosporine, and lovastatin [2]. Ilicicolin H is a fungal natural product that was first isolated from *Cylindrocladium ilicicola* MFC-870 [3]. It displays potent and broad-spectrum antifungal activities with a novel mechanism of action, by inhibiting the yeast cytochrome bc1 complex at the Qn site (IC_50_ 3–5 nM) [4]. However, because of its strong tendency to bind with plasma protein, ilicicolin H shows marked reduced potency in vivo [5]. Merck Research Lab had dedicated considerable effort to improve its biological activities by chemical and enzymatic derivatization of ilicicolin H, but these efforts met with limited success [5,6,7,8].

**Figure 1:**
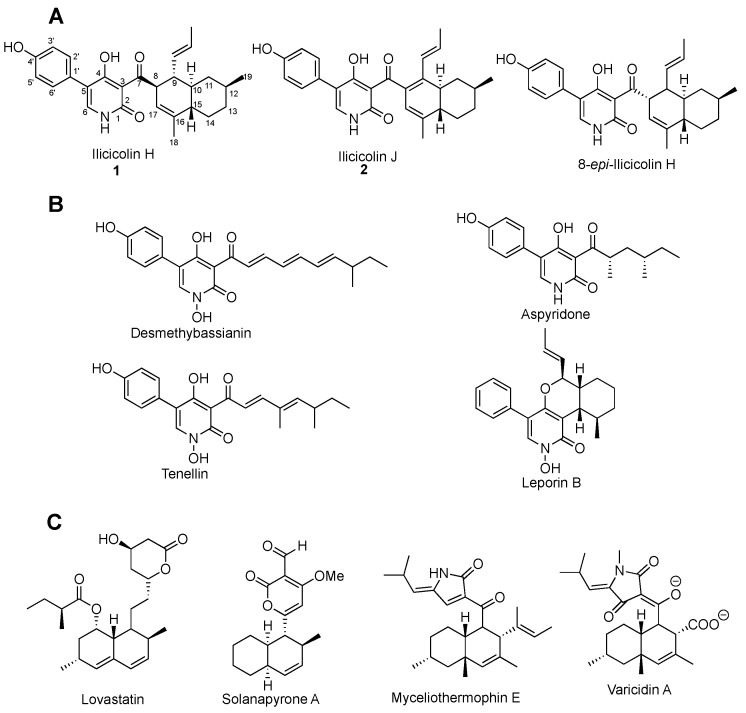
(**A**) Structure of ilicicolin H, ilicicolin J, and 8-*epi*-ilicicolin H. (**B**) Natural products containing 2-pyridone moiety. (**C**) Natural products containing decalin moiety.

Ilicicolin H contains two interesting structural motifs—2-pyridone and decalin (Figure 1). The 2-pyridone motif renders it structurally related to natural products tenellin [9,10,11], desmethylbassianin [12], aspyridone [13,14], and leporine [15,16]. Its decalin moiety resembles that of lovastatin [17,18], solanapyrone [19], myceliothermophin [20], and varicidin [21], thus likely derived from an intramolecular Diels–Alder reaction. The biosynthesis of ilicicolin H has been investigated by stable-isotope labeling, suggesting that it is a polyketide-nonribosomal peptide-type natural product: the backbone is derived from acetyl CoA and tyrosine, while the two methyl groups are from methionine [22]. With the advancement of genomic sequencing and molecular biology, the biosynthetic gene cluster (BGC) of many natural products has been uncovered, and their biosynthesis has been investigated by heterologous production [2,23], and derivatives have been generated by combinatorial biosynthesis [14]. Here, we report our effort on the identification of BGC of ilicicolin H in *Neonectria* sp. DH2, and its heterologous production in *Aspergillus nidulans.* In addition, a new shunt, ilicicolin J, was isolated during the heterologous expression of the BGC. During the course of our study, the ilicicolin H BGC from *Penicillium variabile* was uncovered [24].Those efforts provide a base for future combinatorial biosynthesis of ilicicolin analogs that might solve the plasmid-binding problems.

## 2. Results and Discussion

**Figure 2:**
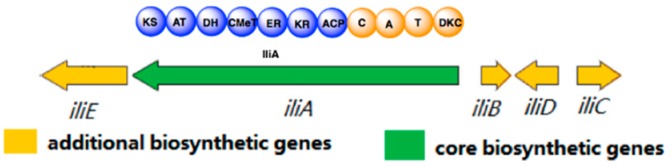
Putative biosynthetic gene cluster of ilicicolin H.

*Neonectria* sp. DH2 is an endophytic fungus that was isolated from *Meconopsis grandis* Prain in Tibet, and it produces a series of secondary metabolites including ilicicolin H (unpublished results). To identify ilicicolin H BGC, we sequenced the whole genome of *Neonectria* sp. DH2 (accession number: RQWH00000000). AntiSMASH [25] analysis of the genomic sequences reveals six BGCs containing polyketide-nonribosomal peptide synthetase (PKS-NRPS), with one BGC shows overall 60% similarity with desmethylbassianin and tenellin BGC. Five genes (*iliA*, *iliB*, *iliC*, *iliD and iliE*) are potentially directly involved in ilicicolin H biosynthesis (Figure 2). IliA is predicated as a PKS-NRPS with a 66% identity to tenellin PKS (TenS) and 68% identity to desmethylbassianin PKS (DmbS). IliA has a typical PKS-NRPS domain arrangement: Ketosynthase (KS), acyl transferase (AT), dehydratase (DH), *C*-methyl transferase (CMeT), ketoreductase (KR), enoyl reductase (ER), and acyl carrier protein (ACP) domains, typical of a highly reducing (HR) PKS; followed by condensation (C), adenylation (A), thiolation (T) domains, and dieckmann cyclase domain (DKC), characteristic of a NRPS module. The ER domain of IliA is probably inactive because it is more related to TenS (containing an inactive ER domain) than mFAS and squalestain tetraketide synthase (containing an active ER domain) (Figure S1). Similar to tenellin and desmethylbassianin biosynthesis [26], a trans-acting ER enzyme, IliB is present in the gene cluster, showing 52% identity to TenC and 53% identity to DmbC, respectively. IliC is predicted as a cytochrome P450, and displays 62% identity to both TenA and DmbA. TenA has been shown to be involved in the ring-expansion of tetramic acid group to pyridones group during tenellin biosynthesis [10], which suggests that IliC might be involved in the formation of pyridone moiety of ilicicolin H. IliD is predicted as a methyltransferase and no homologous gene is found in either tenellin or desmethylbassianin BGCs. Since methyl groups of tenellin and desmethylbassianin are both introduced during the chain elongation stage by CMeT domain of IliA, it is reasonable to assume that this is also true for ilicicolin H. There are at least two *S*-adenosyl-l-methionine (SAM)-dependent enzymes, SpnF [19,27] and LepI [16,28], that have been shown to be involved in Diels–Alder reactions. Therefore, IliD, a putative methyltransferase, might catalyze an intramolecular Diels–Alder reaction to construct the decline moiety of ilicicolin H.

**Figure 3:**
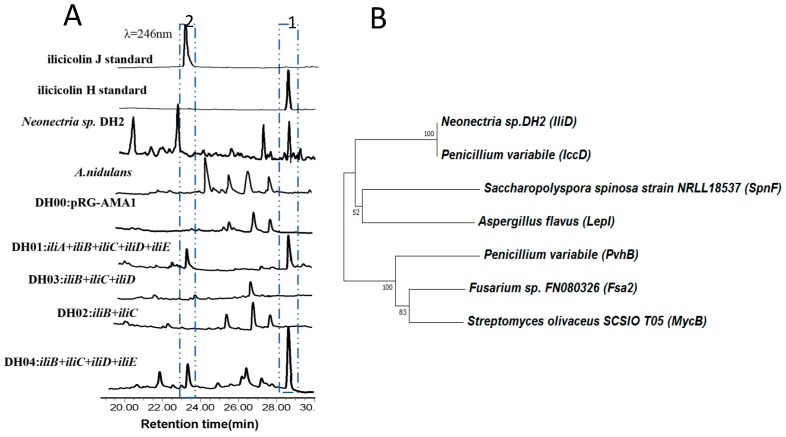
(**A**) HPLC trace of the metabolites of *A. nidulans* strains and *Neonectria* sp. DH2. The peak at 23 min is ilicicolin J and the peak at 29 min is ilicicolin H. (**B**) Phylogenetic analysis of iliD and related pericyclases.

*A. nidulans* is a very successful model organism to identify fungal natural products and their gene clusters [23,29]. Therefore, heterologous expression of the putative ilicicolin H BGC in *A. nidulans* was first attempted. PCR amplification of all the 5 genes, *iliA, iliB, iliC, iliD*, *and iliE* from genomic DNA, followed by Gibson assembly in *E. coli* furnished the expression plasmid pDH1. The plasmid was then transformed into *A. nidulans* and the resulting strain was cultured for 10 days. The organic extract was analyzed by HPLC, and two new peaks were identified compared to strain with an empty plasmid (Figure 3a). The peak at 29 min contains compound **1** (0.55 mg/L) with same molecular formula (C_27_H_31_NO_4_) as ilicicolin H, and co-injection suggested that it is ilicicolin H. The mass spectra of the compound **2** (0.21 mg/L) at 23 min is [M + H]^+^ of 432.2264 *m/z*, suggesting that it is a ilicicolin derivative with one extra degree of unsaturation. To confirm the structure of both compounds, we fermented 5 L of the stain, and detailed NMR analysis confirmed that compound **1** is indeed ilicicolin H. Compound **2** and ilicicolin H have very similar ^1^H- and ^13^C-NMR spectra (Table S4), except for the absence of two methine groups (C-8 and C-9), which were replaced by two olefinic carbons. The additional double bound could be adjacent to the keto carbonyl (C-7) to form a conjugated system, which led to the noticeable change of the carbon chemical shift on the keto carbonyl from 209.0 ppm for ilicicolin H to 195.7 ppm for **2**. The gross structure of **2** was further established by the two-dimensional NMR data. Thus, compound **2** was elucidated as ilicicolin J. Previous efforts on derivatization of ilicicolin H always preserved the C8-stereochemistry, probably assuming it is important for the biological activities. Indeed, 8-*epi*-ilicicolin H (Figure 1A) with an opposite stereoconfiguration shows 100-fold reduction in antifungal activities. Surprisingly, we found that ilicicolin J, with C8-position stereochemistry eliminated, displays comparable antifungal activity with ilicicolin H (MIC 6.3 μg/mL). Our result indicates that future structure-activity relationship (SAR) studies of this class of antifungal reagents can be based on a simpler structure, ilicicolin J.

To investigate the individual function of the five biosynthesis-related genes, another four plasmids, pDH2 (harboring *iliA* and *iliB* gene), pDH3 (harboring *iliA*, *iliB* and *iliC* gene), pDH4 (harboring *iliA*, *iliB*, *iliD* gene) and pDH5 (harboring *iliA*, *iliB*, *iliC and iliD* gene) were constructed. Both ilicicolin H and J were produced by *A. nidulans* strain containing plasmid pDH5, suggesting that the putative oxidoreductase IliE is not directly involved in ilicicolin biosynthesis. During the preparation of this manuscript, Zhuan Zhang et al. reported that a BGC from *Penicillium variabile*, is also responsible for ilicicolin H biosynthesis [24]. Interesting, they showed that without IccE (67% identity with IliE), an epimerase, 8-*epi*-ilicicolin H was produced. Our results indicated that the two fungi might employ different mechanism to form antifungal agent ilicicolin H.

Surprisingly, the strains containing plasmids pDH2, pDH3, pDH4 did not produce any detectable amount of ilicicolin derivatives or biosynthetic intermediates (more than 20 transformants of each construct were screened), which is in contrast to the previous biosynthetic study of tenellin. The *iliC* homolog in tenellin BGC in *Beauveria bassiana*, TenA, has been knockout to test its function [10]. The Knockout *B. bassiana* strain produced a tenellin biosynthetic intermediates, pretenellin-A, and cell-free extracts containing TenA successfully converted the tetramic acid moiety to pyridones. Therefore, we had expected some “preilicicolin” to be produced by either *A. nidulans* strain containing pDH2, pDH3, and pDH4. Similar experiments were performed by Zhuan Zhang et al. and ilicicolin H biosynthetic intermediates were indeed isolated [24]. In their constructs, strong promoters such as PglaA and Pgpda, were placed in front of each biosynthetic genes. However, we have only relied on the ilicicolin H BGC native promoters, therefore, the yield of biosynthetic intermediates might too low to be detected.

Many biosynthetic pericyclases have been uncovered recently [30]. The first enzyme catalyze a solely Diels–Alder reaction is SpnF, which is involved in the spinosyn A biosynthesis, although it was originally annotated as a methyltransferase [19]. Another pericyclases is the recently discovered multifunctional LepI, which was also annotated as a methyltransferase [16]. It catalyzes a cascade of reaction include an intramolecular Diels–Alder reaction, a hetero-Diels–Alder reaction and a retro-Claisen rearrangement. A number of stand-alone decalin forming Diels–Alderases have been reported, such as MycB [20], Fsa2 [31], and PvhB [21]. In the ilicicolin H biosynthesis, IliD is the most possible candidate to catalyze a Diels–Alder reaction to form the decalin moiety. Phylogenetic analysis suggesting that IliD is related to the newly identified Diels–Alderase IccD (Figure 3b). IccD catalyzed the Diels–Alder reaction to form 8-*epi*-ilicicolin H. IliD only show 52% identity with IccD, so it is still possible that IliD directly forms ilicicolin H instead. Expression of IliD in *E. coli* was successful (Figure S2), however, being unable to obtain linear biosynthetic intermediates hindered our effort toward in vitro characterization of IliD function. Sequence alignment shows that IliD displays low sequence homology with SpnF (24% identity) and LepI (19% identity), and most of the homology sequence are located around the SAM-binding site DXGXGXG. Interestingly, we find the SAM-binding site of IliD and SpnF are both located close to *N*-terminus, while that of LepI is close to its *C*-terminus.

**Figure 4:**
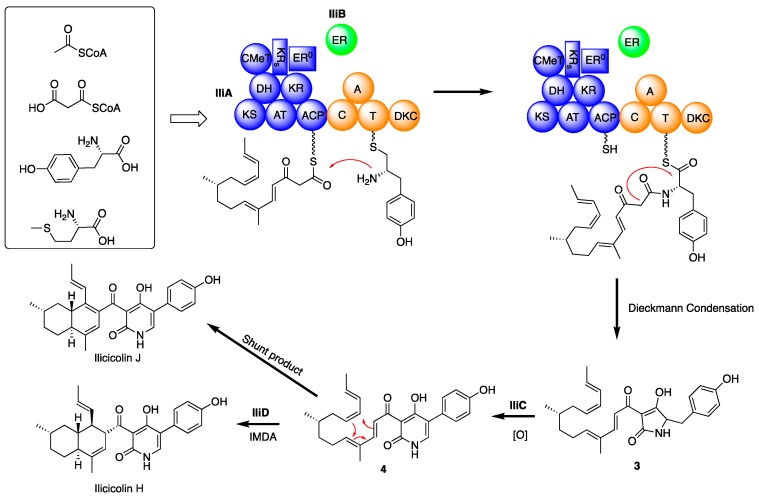
Proposed biosynthetic pathway for ilicicolin H and ilicicolin J.

Based on the above information, we propose the following biosynthetic pathway for ilicicolin H (Figure 4). First, IliA, the PKS-NRPS, assembles the backbone of ilicicolin H. The PKS portion and trans-acting ER domain (IliB) work together to construct an octaketide, and two methyl group is introduced by CMeT domain during the chain assembly. The nascent chain is then condensed with tyrosine, catalyzed by the C domain, and the resulting PKS-NRPS hybrid is offloaded by the DKC domain to form an advanced biosynthetic intermediate **3** with a tetramic acid group. However, we were unable to detect **3** by co-expression of *iliA* and *iliB*, suggesting that IliC might be involve in the chain assembly or offloading processes. Intermediate **3** undergoes a ring expansion process, catalyzed by IliC to furnish intermediate **4**, which subsequence cyclizes to form ilicicolin H, possibly catalyzed by the putative Diels–Alderase IliD. We found expression host cannot convert H to J (Figure S3), thus, the formation of ilicicolin J is still unclear. It is possible the ilicicolin J is a shunt product of the Diels–Alder reaction.

## 3. Materials and Methods

### 3.1. General Experimental Procedure

NMR spectra were obtained on a Bruker AVANCE 400 MHz (Bruker, Switzerland). HR-ESIMS data were measured on a Thermos LCQ DECA XP plus mass spectrometer (Thermos Scientific, Waltham, MA, USA). LC-MS analyses were done in maXis impact spectrometer (Bruker, Germany). HPLC was performed on a Waters 1525 system equipped with a Waters 2998 photodiode array detector.

### 3.2. Strains, Plasmids, and Culture Conditions

*Neonectria* sp. DH2 (Taxonomy ID:1735992), an endophytic fungus, was isolated from *Meconopsis grandis* Prain in Tibet. *Neonectria* sp. DH2 was grown on the PDB medium (300 g/L potato, 20 g/L glucose) or agar plates at 20 °C. To isolated ilicicolin H, *Neonectria* sp. DH2 was incubated in 120 of 1 L flask containing solid rice medium at 20 °C for 28 days. The resulting culture was extracted three times with MeOH to give a crude extract. Then crude extract was then purified by reversed-phase HPLC to get 30 mg of ilicicolin H. *Escherichia coli* strain DH5α were used for routine cloning, which were grown in liquid LB medium or solid medium with 2% agar at 37 °C. *Aspergillus nidulans*, the heterologous host, was grown in GMM medium (10 g/L glucose, 5% 20x nitrate salts, 1% 1000x trace elements, 0.05% uridine, and 0.05% uracil). The antibiotics apramycin were supplemented when required. Fermentation medium consists of 1% glucose, 5% 20x nitrate salts, 1% 1000x trace elements and 1% yeast extract. All the primers and plasmids used in this work are listed in Tables S1 and S2.

### 3.3. Isolation and Manipulation of DNA

Mycelia were collected from the medium for DH2, and lyophilized on FreeZone Freeze Dry Systems (Labconco) for overnight. After grinding the mycelia in liquid nitrogen with a mortar, gDNA was extracted by QIAGEN^®^ Genomic DNA extraction kit (Transgen, Germany) according to the standard operating procedure provided by the manufacturer. Restriction endonucleases were used according to manufacturer’s recommendations (Takara, Japan). The genome of *Neonectria* sp. DH2, was sequenced on Sequel Sequencing Kit 2.1 (Pacific Biosciences, Menlo Park, CA, USA). The assembly genome is approximately 45.8 Mbp, including 43 contigs. The longest contig is 5.08 Mbp and the N50 length is 189,989 bp.

### 3.4. Construction of Recombinant Plasmids

To construct plasmid pDH1, iliA-E were amplified by PCR by premier ABCDE-1F, ABCDE-1R, ABCDE-2F, ABCDE-2R, ABCDE-3F, and ABCDE-3R. The three overlapping DNA fragments and NotI/KpnI-digested pRG-AMA1 expression vector were assembled by NEBuiler 173^®^ HiFi DNA Assembly Kit (NEB, UK) and then transformed into *E. coli* DH5α. The correct colonies were checked by PCR and minipreped to get pDH1. The plasmid pDH2, pDH3, pDH4 and pDH5 were generated following the same protocol of pDH1.

### 3.5. Transformation of A. nidulans

PEG-mediated protoplast transformation was employed to construct the *A. nidulans* transformant strain. Spore suspension (100 μL) of the parent strain was inoculated in 10 mL GMM medium for 8 h. The cell wall was then removed using the Yatalase enzyme system (2% Yatalase, 5% lysing enzyme from Trichoderma harzianum) at 30 °C for 3–4 h. The protoplasts were collected and washed with trapping buffer (0.6 M sorbitol, 50 mM CaCl_2_·2H_2_O, 35 mM NaCl, 10 mM Tris-HCl, pH 7.5). Plasmids (10 μg) and 200 μL protoplast suspension were gently mixed and placed on ice for 30 min. Subsequently, 1.35 mL PEG solution (60% PEG4000, 50 mM CaCl_2_·2H_2_O, 10 mM Tris-HCl, pH 7.5) was added. After 20 min at the room temperature, 5 mL STC buffer was added, and spread on the under-layer medium, which was covered with the upper-layer medium. The selective medium, SMM medium, contains 1% glucose, 5% 20x nitrate salts, 0.1% 1000x trace elements, 1.2 M sorbitol, and 0.1% stock pyridoxin HCl. And the transformants could be obtained after incubation at 28 °C for 3–5 days and confirmed via PCR (Figure S1). About 20 transformants were screened for new compound production, and at least one transformants were sequenced with sanger sequencing to confirm no mutations in the sequences.

### 3.6. Isolation and Purification

The supernatant of the fermentation broth (300 mL) was extracted with ethyl acetate, and the mycelia was extract with methanol. The combined crude extract was analyzed by HPLC with a Ultimate XB-C18 column (5 μm, 4.6 mm × 250 mm; Welch, Shanghai, China) with a linear gradient from 75% to 25% B/A in 20 min (phase A: H_2_O; phase B: ACN). The culture broth of a scaled-up culture (5 L) was extracted with ethyl acetate and mycelia was extract with methanol. The combined crude extract was fractionated by normal phase chromatography on a prepacked silica cartridge (Haiyang Chemical, Qingdao, China, 200–300 mesh) using petroleum ether, ethyl acetate and methanol as mobile phase. The fractions were then purified by Semi-preparative HPLC on Ultimate XB-C18 column (5 μm, 4.6 × 250 mm; Welch) to get Ilicicolin J (3 mg) and Ilicicolin H (1 mg). The HPLC analysis of ilicicolin H and ilicicolin J transformation was done with slightly different conditions, with isocratic elution of 90% B/A (phase A: H_2_O; phase B: ACN).

### 3.7. Overproduction of iliD in E. coli

The PCR fragment containing the entire coding sequence of *iliD* was amplified from cDNA. Each PCR fragment was cloned into vector pET28a, which was subsequently digested by appropriate restriction enzymes. The NdeI-Bg1 II fragment for *iliD* was ligated into the NdeI-Bg1 II linearized pET28a to yield the *E. coli* expression vector pDH06. *E. coli* BL21 harboring the plasmid pDH06 were cultivated in 500 mL Erlenmeyer flasks containing 100 mL liquid LB medium supplemented with appropriate antibiotics and grown at 37 °C to an absorption at 600 nm OD of 0.6. Isopropyl thiogalactoside (IPTG) was added to a final concentration of 0.1 mM and the cells were cultivated for further 16 h at 16 °C for induction. Pellets were collected by using centrifuge and resuspended in lysis buffer (10 mM imidazole, 50 mM NaH_2_PO_4_, 300 mM NaCl, pH 8.0) and lysed on ice by sonication. The lysate was centrifuged at 13,000× *g* for 30 min at 4 °C to remove the cellular debris. The purification of the recombinant His-tagged fusion protein by affinity chromatography with Ni-NTA agarose resin was carried out according to the manufacturer’s instructions.

### 3.8. Antifungal Test of Compounds towards Candida Albicans ATCC10231

The antifungal activities of ilicicolin H and ilicicolin J against *Candida albicans* fungal strains were tested by the minimum inhibitory concentration (MIC) method [32] The *Candida albicans* ATCC10231 strains were seeded in PDB medium and then incubated at 28 °C for 18 h. After dilution with PDB broth to 5 × 10^4^ cells/mL, the 200 μL of cell suspension was dispensed into 96-well plates. Different concentrations of sample solutions in DMSO were dispensed into 96-well plates. The PDB broth was used as a blank, and the DMSO and fluconazole were used as a negative and a positive control, respectively. The growth of *Candida albicans* strains was measured after 12–18 h of incubation at 28 °C on a microplate reader at the wavelength of 600 nm. Each assay was performed in triplicate. The MIC of ilicicolin H and ilicicolin J are both 6.3 μg/mL (positive control fluconazole is 3.13 μg/mL).

## 4. Conclusions

In summary, we successfully identified the BGC of ilicicolin H, a potent and broad-spectrum antifungal agent, by genomic sequencing a producing strain. We have shown that four genes are directly involved in ilicicolin H biosynthesis by heterologous expression in *A. nidulans.* IliD, which harbors a SAM-binding site in the *N*-terminus, similar as SnpF, is proposed to catalyze a Diels–Alder reaction to generate the decline moiety. A new natural product, ilicicolin J was isolated and it displays comparable antifungal activity with ilicicolin H, suggesting future SAR studies toward better in vivo antifungal biological activities could be based on a simpler structure.

## Figures and Tables

**Table:** 

Protein	Size (aa)	Proposed Function	Tenellin BGC Homolog	Similarity/Identity (%)	DMBA BGC Homolog	Similarity/IDentity (%)
IliA	2000	PKS-NRPS	tenS	78/66	dmbS	80/68
IliB	381	ER	tenC	67/52	dmbC	67/53
IliC	505	cytochrome P450	tenA	78/63	dmbA	78/63
IliD	244	methyltransferase	-	-	-	-
IliE	766	NADH:Flavin Oxidoreductase	-	-	-	-

## Supplementary Materials

The following are available online. Figure S1. Phylogenetic analysis of ER domain; Figure S2. The sequence alignment of ER domain between iccA and iliA; Figure S3. SDS-PAGE analysis of iliD protein expression; Figure S4. The transformation of ilicicolin H and ilicicolin J; Figure S5. Schematic diagram of Gibson assembly; Table S1. Primers used for constructing recombinant plasmids; Table S2. Strains used in this work [23,33]; Table S3. ilicicolin H gene cluster in *Neonectria* sp. DH2 comparinging with gene cluster in *Penicillium variabile*; Figure S6. Synteny map of ilicicolin H gene clusters in *Neonectria* sp. DH2 and *Penicillium variabile*; Figure S7. The antifungal activities of ilicicolin H and ilicicolin J against *Candida albicans* fungal strains; Table S4. ^1^H and ^13^C-NMR spectroscopic data of Ilicicolin J (400 and 100 MHz, DMSO-*d*_6_) and Ilicicolin H (400 and 100 MHz, DMSO-*d*_6_); Table S5. NMR chemical shifts of Ilicicolin H in DMSO-*d_6_* and reported [34] Ilicicolin H in Acetonitrile-*d_3_*; Figure S8. The ^1^H-NMR (400 MHz, DMSO-*d*_6_) and ^13^C-NMR (100 MHz, DMSO-*d*_6_) spectrum of the Ilicicolin J; Figure S9. The ^1^H-NMR (400 MHz, DMSO-*d*_6_) and ^13^C-NMR (100 MHz, DMSO-*d*_6_) spectrum of the Ilicicolin H; Figure S10. The HMBC spectrum of the Ilicicolin J; Figure S11. The HSQC spectrum of the Ilicicolin J; Figure S12. The HMBC spectrum of the Ilicicolin H; Figure S13. The HSQC spectrum of the Ilicicolin H; Figure S14. The HRESIMS spectrum of the Ilicicolin J; Figure S15. The HRESIMS spectrum of the Ilicicolin H.
