# Supplementary material for: Heterologous Expression of Ilicicolin H Biosynthetic Gene Cluster and Production of a New Potent Antifungal Reagent, Ilicicolin J
Source: Molecules. 2019 Jun 18;24(12):2267. doi: 10.3390/molecules24122267 (PMC6631495; doi:10.3390/molecules24122267)
Supplement: Supplementary file 1 [file molecules-24-02267-s001.pdf]

Heterologous expression of ilicicolin H biosynthetic gene cluster and production of a new potent antifungal reagent, ilicicolin J

Xiaojing Lin[1,2], Siwen Yuan[1], Senhua Chen[1,3], Bin Chen[1,3], Xu Hui[2], Lan Liu[1,3], Huixian Li[*1], Zhizeng Gao[*1,3]

[1]*School of Marine Sciences, Sun Yat-sen University, Guangzhou 510006, China.*
E-mail: gaozhizeng@mail.sysu.edu.cn, lihx46@mail.sysu.edu.cn
[2]*Research Center of Chinese Herbal Resource Science and Engineering, Guangzhou University of Chinese Medicine, Guangzhou 510006, China*
[3]*Southern Laboratory of Ocean Science and Engineering, Zhuhai 519080, People's Republic of China*
*Correspondence: gaozhizeng@mail.sysu.edu.cn; lihx46@mail.sysu.edu.cn

List of Contents

Figure S1. Phylogenetic analysis of ER domains

Figure S2. The sequence alignment of ER domain between iccA and iliA.

Figure S3. SDS-PAGE analysis of iliD protein expression.

Figure S4. The transformation of ilicicolin H and ilicicolin J.

Figure S5. Schematic diagram of Gibson assembly

Table S1. Primers used for constructing recombinant plasmids.

Table S2. Strains used in this work.

Table S3. ilicicolin H gene cluster in *Neonectria sp.* DH2 comparing with gene cluster in *Penicillium variabile.*

Figure S6. Synteny map of ilicicolin H gene clusters in *Neonectria* sp. DH2 and *Penicillium variabile*.

Figure S7. The antifungal activities of ilicicolin H and ilicicolin J against *Candida albicans* fungal strains

Table S4. ^{1}H and ^{13}C NMR spectroscopic data of Ilicicolin J (400 and 100 MHz, DMSO-d_6) and Ilicicolin H (400 and 100 MHz, DMSO-d_6).

Table S5. NMR chemical shifts of Ilicicolin H in DMSO-d_6 and reported Ilicicolin H in Acetonitrile-d_3.

Figure S8. The ^{1}H NMR (400 MHz, DMSO-d_6) and ^{13}C NMR (100 MHz, DMSO-d_6) spectrum of the Ilicicolin J.

Figure S9. The ^{1}H NMR (400 MHz, DMSO-d_6) and ^{13}C NMR (100 MHz, DMSO-d_6) spectrum of the Ilicicolin H.

Figure S10. The HMBC spectrum of the Ilicicolin J.

Figure S11. The HSQC spectrum of the Ilicicolin J.

Figure S12. The HMBC spectrum of the Ilicicolin H.

Figure S13. The HSQC spectrum of the Ilicicolin H.

Figure S14. The HRESIMS spectrum of the Ilicicolin J.

Figure S15. The HRESIMS spectrum of the Ilicicolin H.

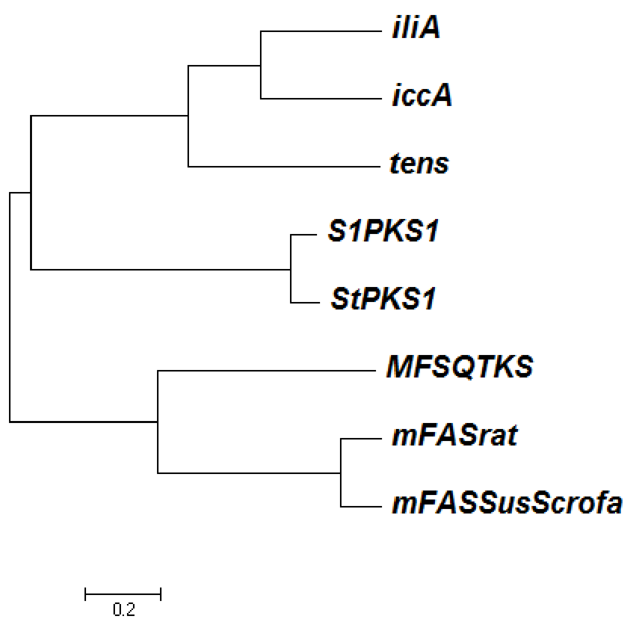

FigureS1. Phylogenetic analysis of ER domains. mFAS: mammalian fatty acid synthase; Tens: tenellin synthetase polyketide synthase; StPKS1: S. tenacellus polyketide synthase; S1 PKS: S. lutea F23523 polyketide synthase; MFSQTKS: squalestatin tetraketide synthase; iliA: ilicicolin H synthetase in *Neonectria sp.* DH2; iccA: ilicicolin H synthetase in *Penicillium variabile.* ER domain sequence were exctracted and aligned using ClustalX. The alignment was then subjected to boot-strapped phylogenetic analysis using the Neighbour-joining analysis in MAGE 7.

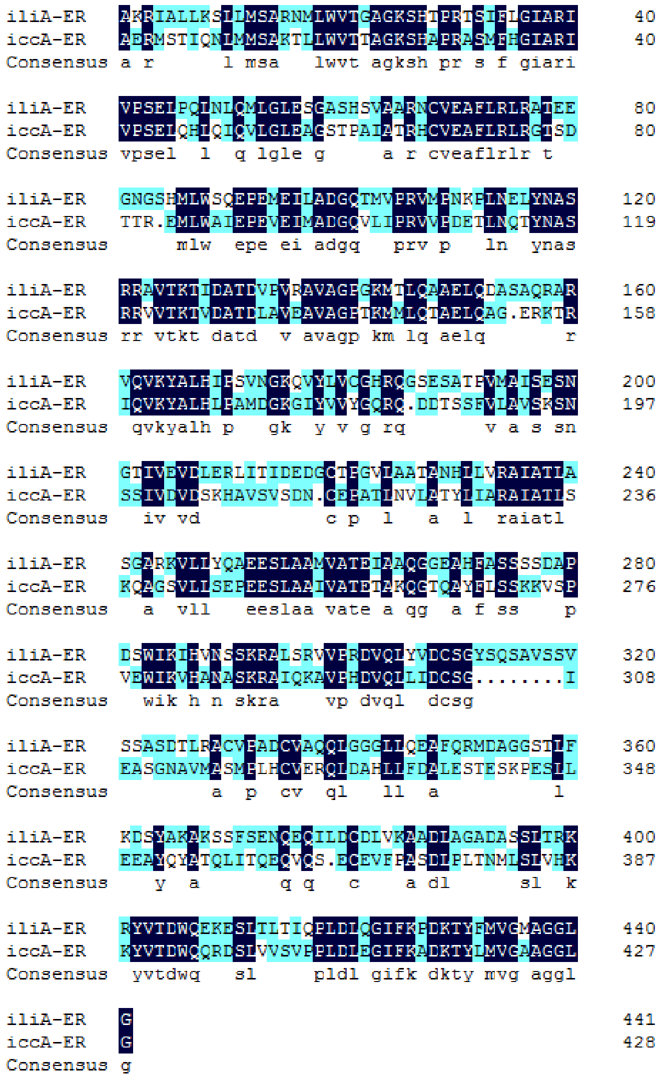

Figure S2. The sequence alignment of ER domains between iccA and iliA.

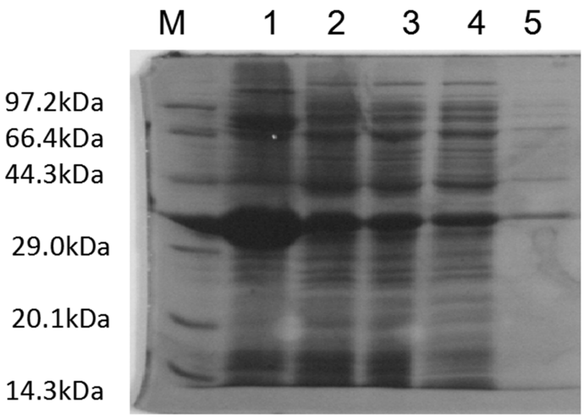

FigureS3. SDS-PAGE analysis of iliD protein expression. M:protein marker; 1:iliD cell debris; 2:iliD supernatant; 3: Flow through 4:Washed buffer; 5:Purified iliD

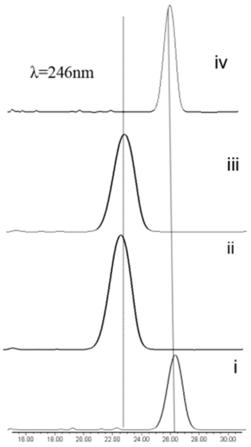

Figure S4. The transformation of ilicicolin H and ilicicolin J. i) Ilicicolin H standard. ii) Ilicicolin J standard. iii) Ilicicolin J was added to WT *A. nidulans* culture, and incubate for 5 days. No ilicicolin H was formed. iv) Ilicicolin H was added to WT *A. nidulans* culture, and incubate for 5 days. No ilicicolin J was formed.

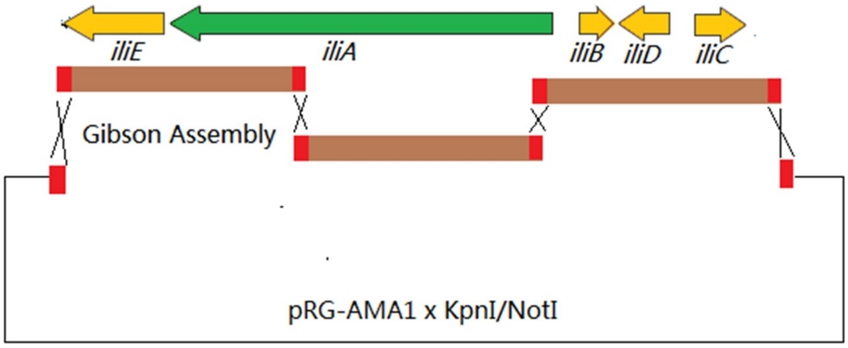

Figure S5. Schematic diagram of Gibson assembly. The same color parts represent the same sequences for homologous recombination.

Table S1. Primers used for constructing recombinant plasmids

Primer	Sequence (5′ to 3′)	Usage
ABCDE-1F	GAATTCGAGCTCGGTACCCGGGGATTCATCCTTCTCTAAGAGCCG	Cloning of *iliA-E* to construct plasmid pDH01 from *Neonectria sp.* DH2
ABCDE-1R	GGCGACACCGTGATCCAGGTTTGCACGTGG	
ABCDE-2F	AGCTGTGCCGCCTCCTTCTTCTTCATG	
ABCDE-2R	TGCTGGCCTCGACCCGATCAAGGAC	
ABCDE-3F	GTACTGAGGCCGGTCCTTGATCGGGTCGAG	
ABCDE-3R	AAAAAATAAGCTTGCATGCGCGGCCCACCCAATCGCCACGCACGCTACAG	
BCDE-1F	GAATTCGAGCTCGGTACCCGGGGATCAATGCTCCCTGTAAACAGATATAC	Cloning of *iliA-D* to construct plasmid pDH02 from *Neonectria sp.* DH2
BCDE-1R	GTCTCAAGTGTCTCATCTGCCTCGG	
BCDE-2F	CCGAGGCAGATGAGACACTTGAGAC	
BCDE-2R	AGCGAAAAGAATGACCCACCCCTCC	
BCDE-3F	GGAGGGGTGGGTCATTCTTTTCGCT	
BCDE-3R	AAAAAATAAGCTTGCATGCGCGGCCACAGCATGTCTTCCACTGACCAATC	
BCD-1F	GAATTCGAGCTCGGTACCCGGGGATCAATGCTCCCTGTAAACAGATATAC	Cloning of *iliA-C* to construct plasmid pDH03 from *Neonectria sp.* DH2
BCD-1R	GGCGACACCGTGATCCAGGTTTGCACGTGG	
BCD-2F	AGCTGTGCCGCCTCCTTCTTCTTCATG	
BCD-2R	TGCTGGCCTCGACCCGATCAAGGAC	
BCD-3F	GTACTGAGGCCGGTCCTTGATCGGGTCGAG	
BCD-3R	AAAAAATAAGCTTGCATGCGCGGCCAGGGCAGGTAATAGTTGCCCTTC	
BC-1F	GAATTCGAGCTCGGTACCCGGGGATCGGTGGCAGTAGGATTGAGATTGAC	Cloning of *iliA-B* to construct plasmid pDH04 from *Neonectria sp.* DH2
BC-1R	GAGTCTGGGGCATCGCATTCGGTTG	
BC-2F	CAACCGAATGCGATGCCCCAGACTC	
BC-2R	CAAAAAATAAGCTTGCATGCGCGGCCTATCACCTGTCCCTGTCAGTCCTCC	
BCE-1F	GAATTCGAGCTCGGTACCCGGGGATCAATGCTCCCTGTAAACAGATATAC	Cloning of *iliA*、*iliB and iliD* to construct plasmid pDH05 from *Neonectria sp.* DH2
BCE-1R	AGGCGTGCTGTTACGAAGACCAT	
BCE-2F	ATGGTCTTCGTAACAGCACGCCT	
BCE-2R	ATATGATATGTGACACTCTCGGT	
BCE-3F	ACCGAGAGTGTCACATATCATATAGCCTTTACCTCGCCCACTTACA	
BCE-3R	AAAAAATAAGCTTGCATGCGCGGCCCGCCACGCACGCTACAGTACTAC	
Me-F	CTTTCATATGACTTCGACGGAAGCC	Cloning of *iliD* to construct plasmid pDH06 from *Neonectria sp.* DH2
Me-R	ACAGATCTATCTACTTGGTCTTCGC	

Table S2. Strains used in this work

strain	Features	Reference/Source
Aspergillus nidulans	Heterologous expression host	Junko Y. *et al*.[1]
DH01	Exconjugant strains with plasmid pDH01	This work
DH02	Exconjugant strains with plasmid pDH02	This work
DH03	Exconjugant strains with plasmid pDH03	This work
DH04	Exconjugant strains with plasmid pDH04	This work
DH05	Exconjugant strains with plasmid pDH05	This work
DH06	Exconjugant strains with plasmid pDH06	This work
Escherichia coli DH5α	General cloning strain	Zhiming, T. *et al*[2].

TableS3. ilicicolin H gene cluster in *Neonectria sp.* DH2 comparing with gene cluster in *Penicillium variabile.*

Protein	Homologs	similarity	identity
IliA	IccA	80%	66%
IliB	IccB	81%	69%
IliC	IccC	80%	67%
IliD	IccD	68%	52%
IliE	IccE	73%	58%

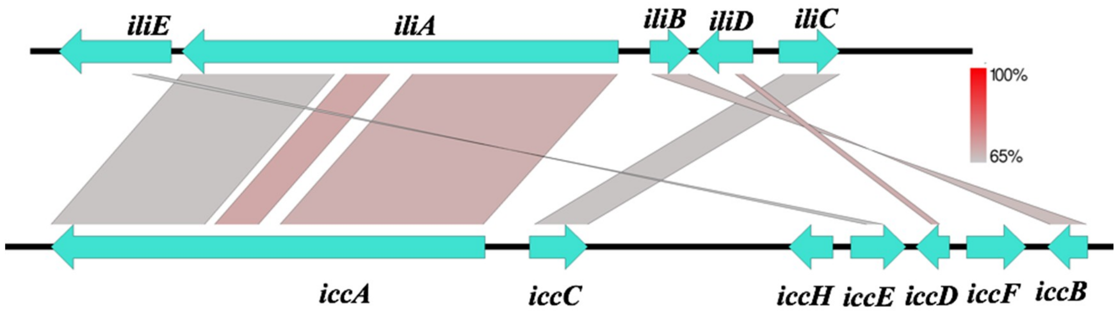

Figure S6. Synteny map of ilicicolin H gene clusters in *Neonectria* sp. DH2 and *Penicillium variabile*.

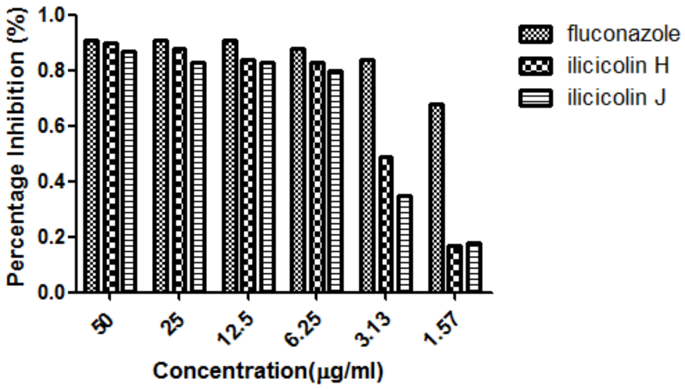

Figure S7. The antifungal activities of ilicicolin H and ilicicolin J against *Candida albicans* fungal strains.

Table S4. ^{1}H (400MHz) and ^{13}C (100MHz) NMR chemical shifts of Ilicicolin H and J in DMSO-d_6

Position		Ilicicolin J			Ilicicolin H	
		δ_H(ppm, *J* in Hz)	δ_C (ppm)		δ_H(ppm, *J* in Hz)	δ_C (ppm)
1	NH			NH		
2	C		157.6	C		161.7
3	C		106.2	C		106.5
4	C		181.1	C		175.9
5	C		108.1	C		112.5
6	CH	7.75 (1H, s)	141.9	CH	7.55 (1H, d, 6.7)	141.3
7	C		195.7	C		209.0
8	C		99.5	CH	4.92 (1H, m)	52.6
9	C		93.1	CH	2.43(1H, app. q,10.1)	44.0
10	CH	2.45(1H, m)	51.3	CH	1.33(1H, m)	43.0
11	CH_2	2.03(1H, m); 0.54 (1H, app. q, 12.1)	39.2	CH_2	1.70 (1H, m); 0.53 (1H, app. q,12.0)	39.4
12	CH	1.40 (1H, m)	32.0	CH	1.12 (1H, m)	32.4
13	CH_2	1.75 (1H, m); 0.98 (1H, m)	35.5	CH_2	1.12 (1H, m); 0.92 (1H, m)	35.2
14	CH_2	2.03 (1H, m); 0.98 (1H, m)	28.8	CH_2	1.96 (1H, m); 0.92 (1H, m)	29.5
15	CH	1.75(1H, m)	44.1	CH	1.70 (1H, m)	44.2
16	C		144.9	C		137.5
17	CH	5.08 (1H, s)	118.1	CH	5.13 (1H, s)	119.9
18	CH_3	1.68 (3H, s)	20.4	CH_3	1.58 (3H, s)	20.9
19	CH_3	0.87 (3H, d, 6.5)	22.6	CH_3	0.85 (3H, d,6.5)	22.8
20	CH	5.51(1H, m)	129.6	CH	5.16 (1H, m)	133.5
21	CH	4.95(1H, m)	126.7	CH	5.25(1H, m)	125.7
22	CH_3	1.47 (3H, d, 6.0)	18.2	CH_3	1.50 (3H, d,6.0)	18.1
1′	C	1.59 (1H, d,12.4)	122.1	C		123.5
2′	CH	7.33 (2H, d,9.5)	128.9	CH	7.21 (2H, d,8.5)	130.4
3′	CH	6.82 (2H, d,8.6)	115.5	CH	6.75 (2H, d,8.5)	115.2
4′	C		157.0	C		157.0
5′	CH	6.82 (2H, d,8.6)	115.5	CH	6.75 (2H, d,8.5)	115.2
6′	CH	7.33 (2H, d,9.5)	128.9	CH	7.21 (2H, d,8.5)	130.4

Table S5. NMR chemical shifts of Ilicicolin H in DMSO-d_6 and reported[3] Ilicicolin H in Acetonitrile-d_3

Position		Reported Ilicicolin H			Ilicicolin H	
		δ_H(ppm, *J* in Hz)	δ_C (ppm)		δ_H(ppm, *J* in Hz)	δ_C (ppm)
1	NH			NH		
2	C		162.9	C		161.7
3	C		108.1	C		106.5
4	C		178.0	C		175.9
5	C		114.8	C		112.5
6	CH	7.40 (1H, s)	141.4	CH	7.55 (1H, d, 6.7)	141.3
7	C		211.0	C		209.0
8	C	4.97 (1H, m)	54.1	CH	4.92 (1H, m)	52.6
9	C	2.48(1H, app. q,10.4)	46.2	CH	2.43(1H, app. q,10.1)	44.0
10	CH	1.23(1H, m)	44.5	CH	1.33(1H, m)	43.0
11	CH_2	1.77(1H, m); 0.58 (1H, app. q, 11.8)	40.6	CH_2	1.70 (1H, m); 0.53 (1H, q ,12.0)	39.4
12	CH	1.38 (1H, m)	33.8	CH	1.12 (1H, m)	32.4
13	CH_2	1.77 (1H, m); 0.97 (1H, m)	36.6	CH_2	1.12 (1H, m); 0.92 (1H, m)	35.2
14	CH_2	2.04 (1H, m); 0.99 (1H, m)	31.0	CH_2	1.96 (1H, m); 0.92 (1H, m)	29.5
15	CH	1.68(1H, m)	45.4	CH	1.70 (1H, m)	44.2
16	C		139.5	C		137.5
17	CH	5.21 (1H, s)	120.9	CH	5.13 (1H, s)	119.9
18	CH_3	1.63 (3H, s)	21.5	CH_3	1.58 (3H, s)	20.9
19	CH_3	0.89 (3H, d, 6.5)	23.4	CH_3	0.85 (3H, d,6.5)	22.8
20	CH	5.21(1H, m)	134.8	CH	5.16 (1H, m)	133.5
21	CH	5.32(1H, m)	127.3	CH	5.25(1H, m)	125.7
22	CH_3	1.53 (3H, d, 6.0)	18.5	CH_3	1.50 (3H, d,6.0)	18.1
1′	C		125.3	C		123.5
2′	CH	7.26 (2H, d,8.6)	131.4	CH	7.21 (2H, d,8.5)	130.4
3′	CH	6.83 (2H, d,8.6)	116.0	CH	6.75 (2H, d,8.5)	115.2
4′	C		157.5	C		157.0
5′	CH	6.83 (2H, d,8.6)	116.0	CH	6.75 (2H, d,8.5)	115.2
6′	CH	7.26 (2H, d,8.6)	131.4	CH	7.21 (2H, d,8.5)	130.4

Reference:

1 J. Yaegashi, B. R. Oakley and C. C. C. Wang, *J. Ind. Microbiol. Biotechnol.* 2014 , 41, 433–442.

2 Z. Tu, G. He, K. X. Li, M. J. Chen, J. Chang, L. Chen, Q. Yao, D. P. Liu, H. Ye, J. Shi and X. Wu, *Electron. J. Biotechnol.* 2005 , **8**, 113–120.

3 Kildgaard. S, Subko. K, Phillips E, Goidts.V, de la Cruz. M et al *Marine Drugs* 2017 , 15, 253

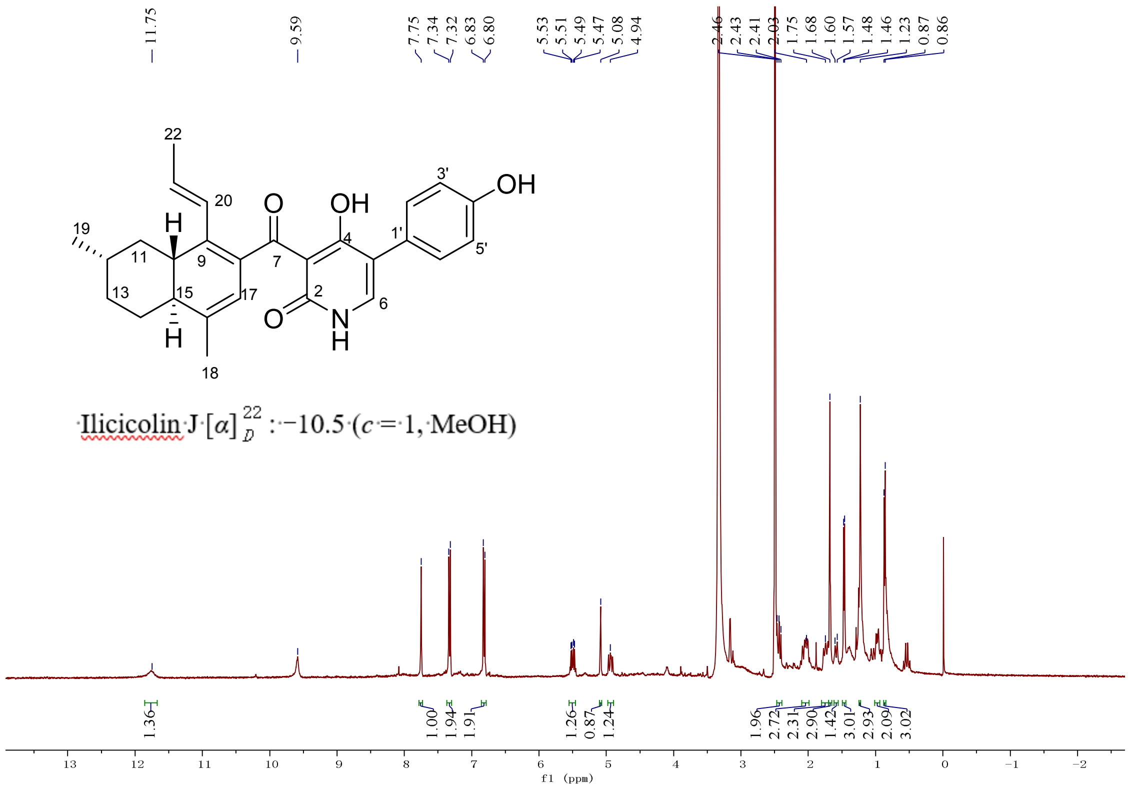

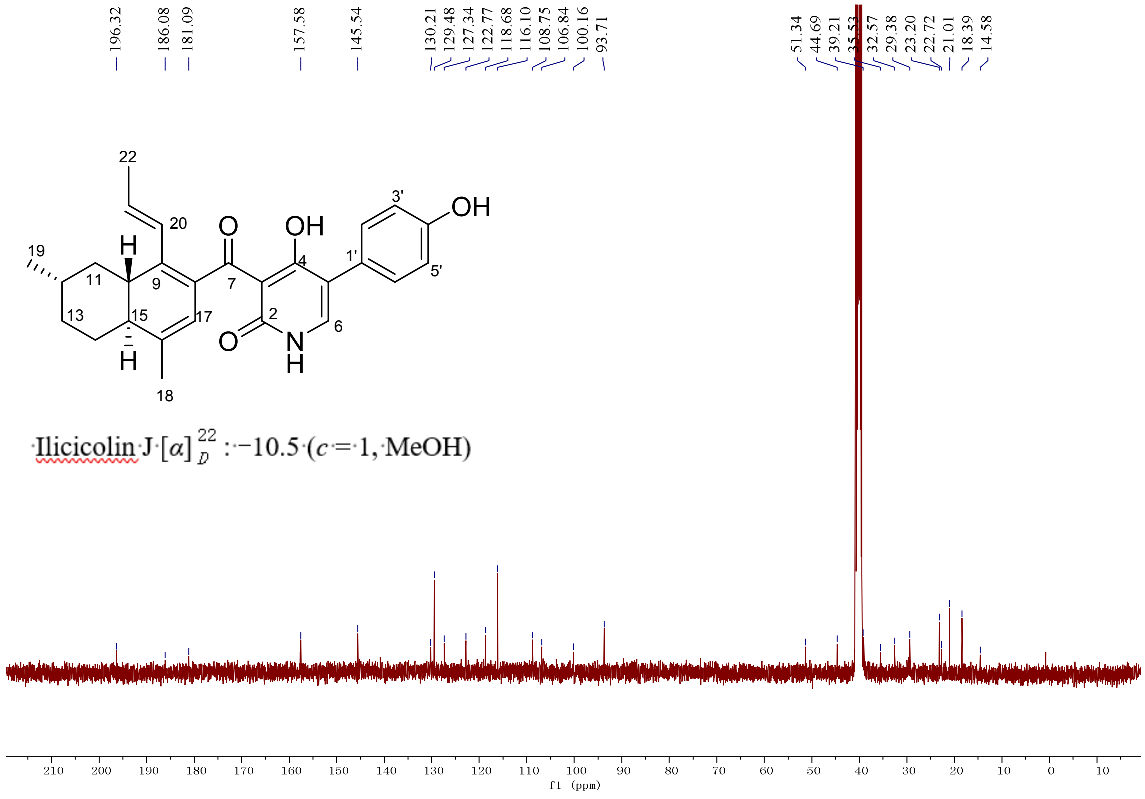

Figure S8. The ^{1}H NMR (400 MHz, DMSO-*d6*) and ^{13}C NMR (100 MHz, DMSO-*d6*) spectrum of ilicicolin J.

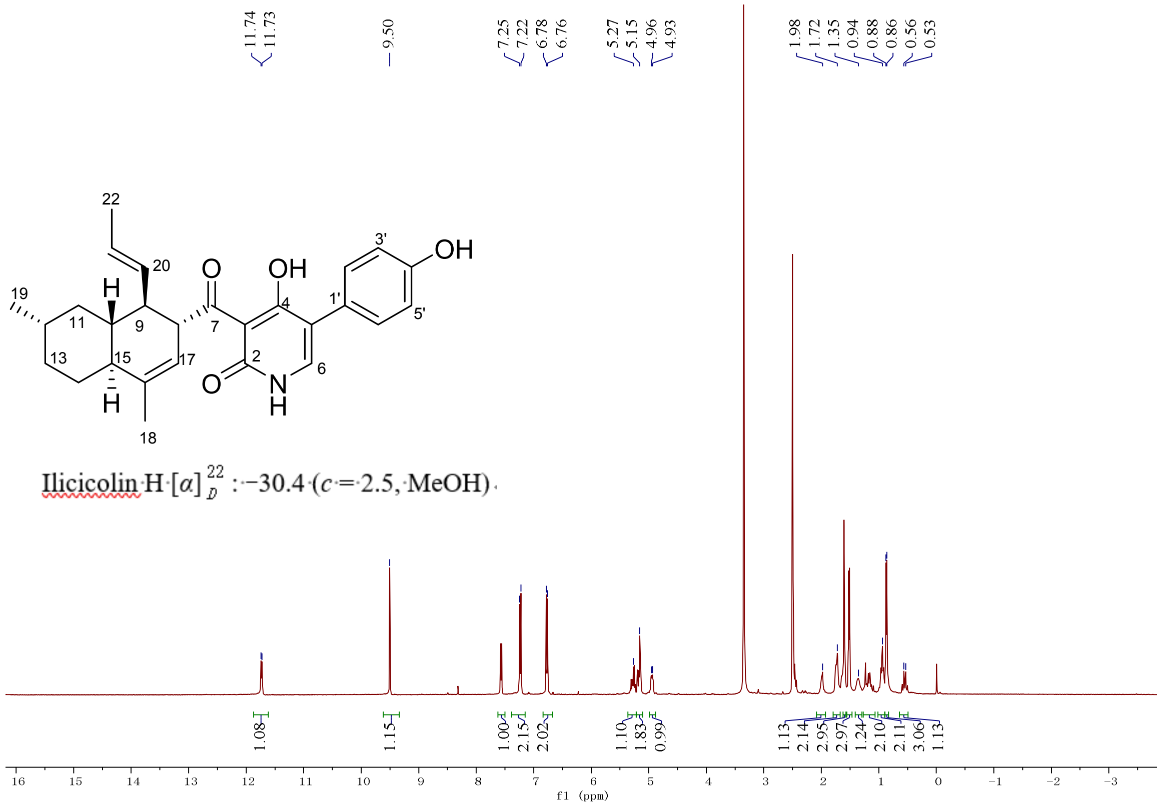

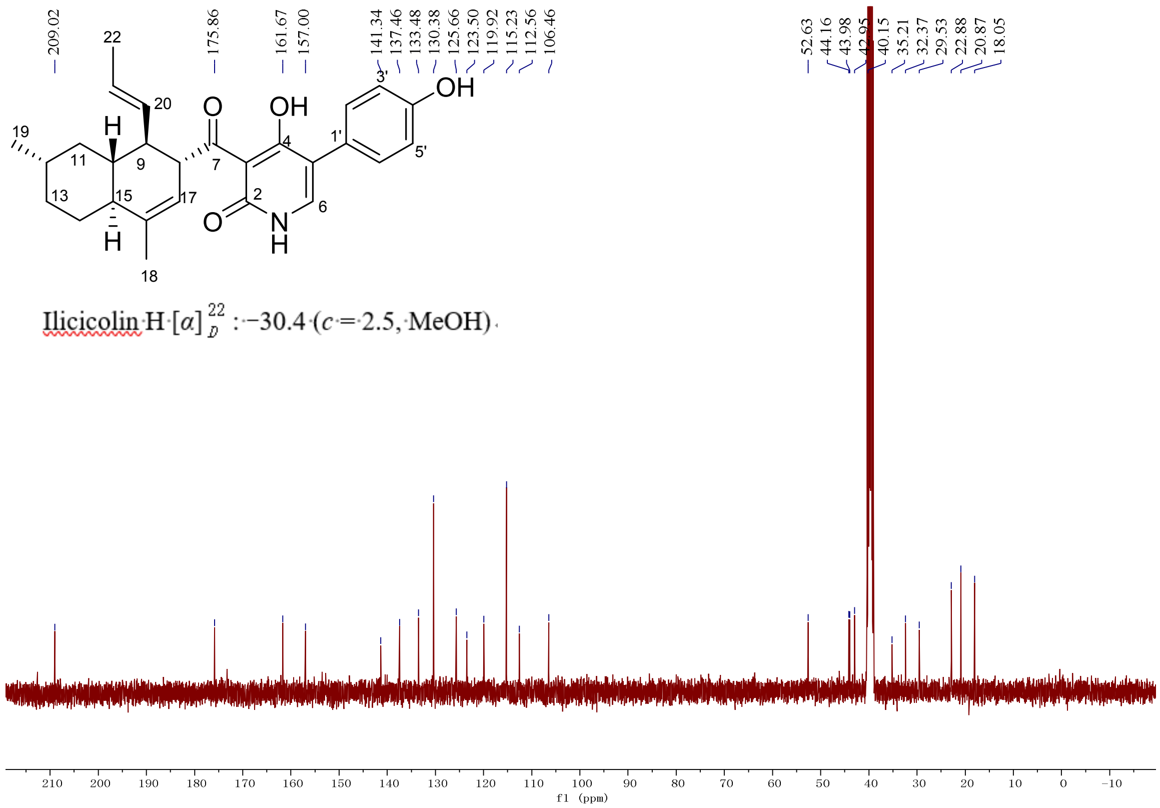

Figure S9. The ^{1}H NMR (400 MHz, DMSO-*d*6) and ^{13}C NMR (100 MHz, DMSO-*d*6) spectrum of ilicicolin H.

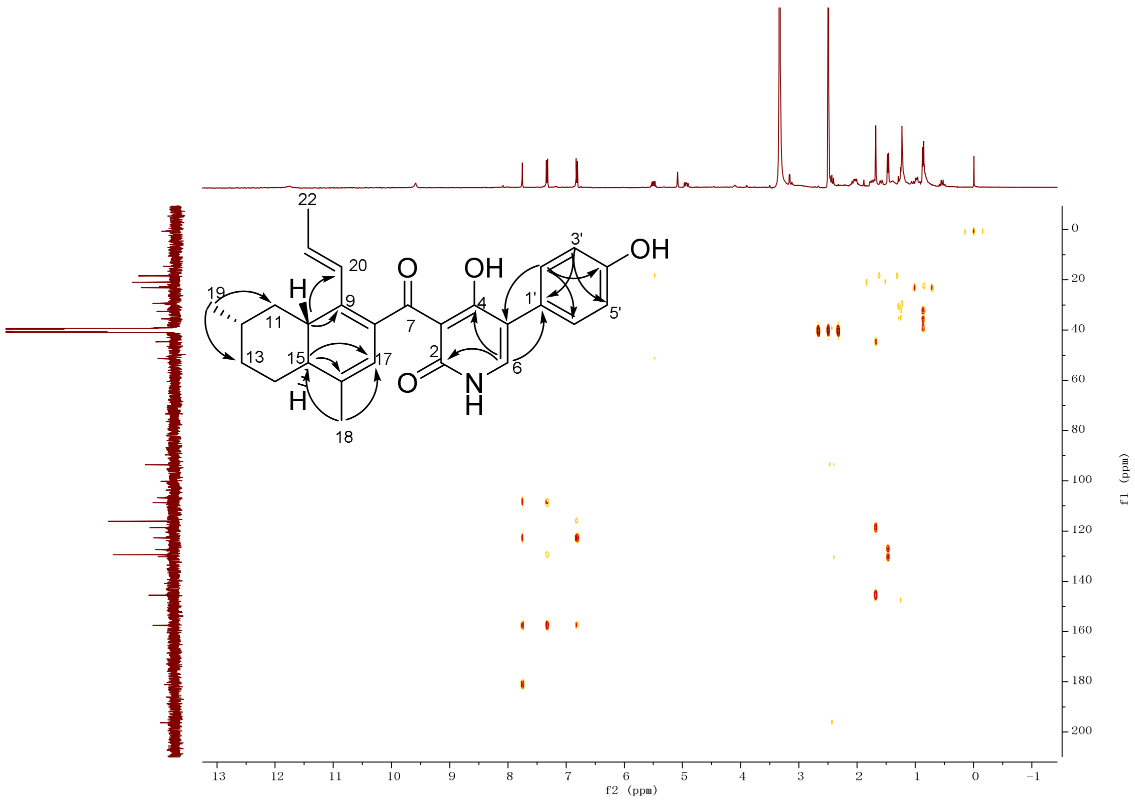

Figure S10. HMBC spectrum of ilicicolin J in DMSO-d_6 at 400 MHz

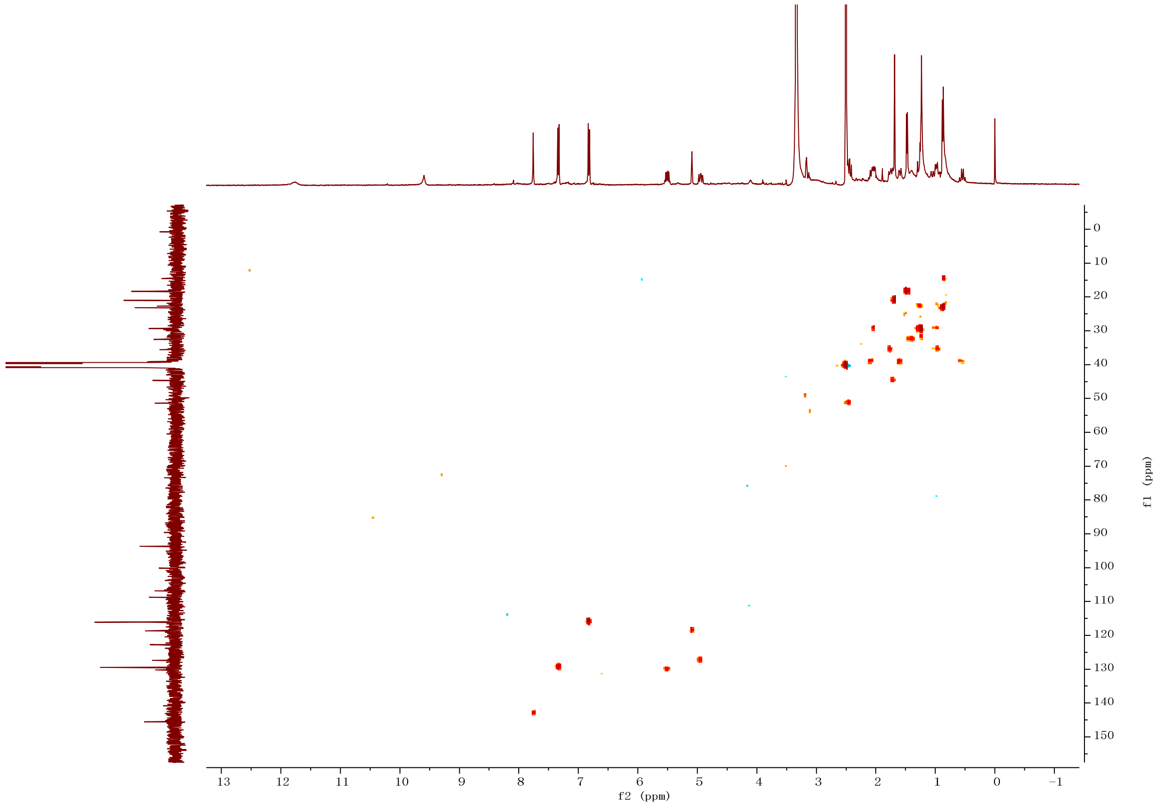

Figure S11. HSQC spectrum of ilicicolin J in DMSO-d_6 at 400 MHz

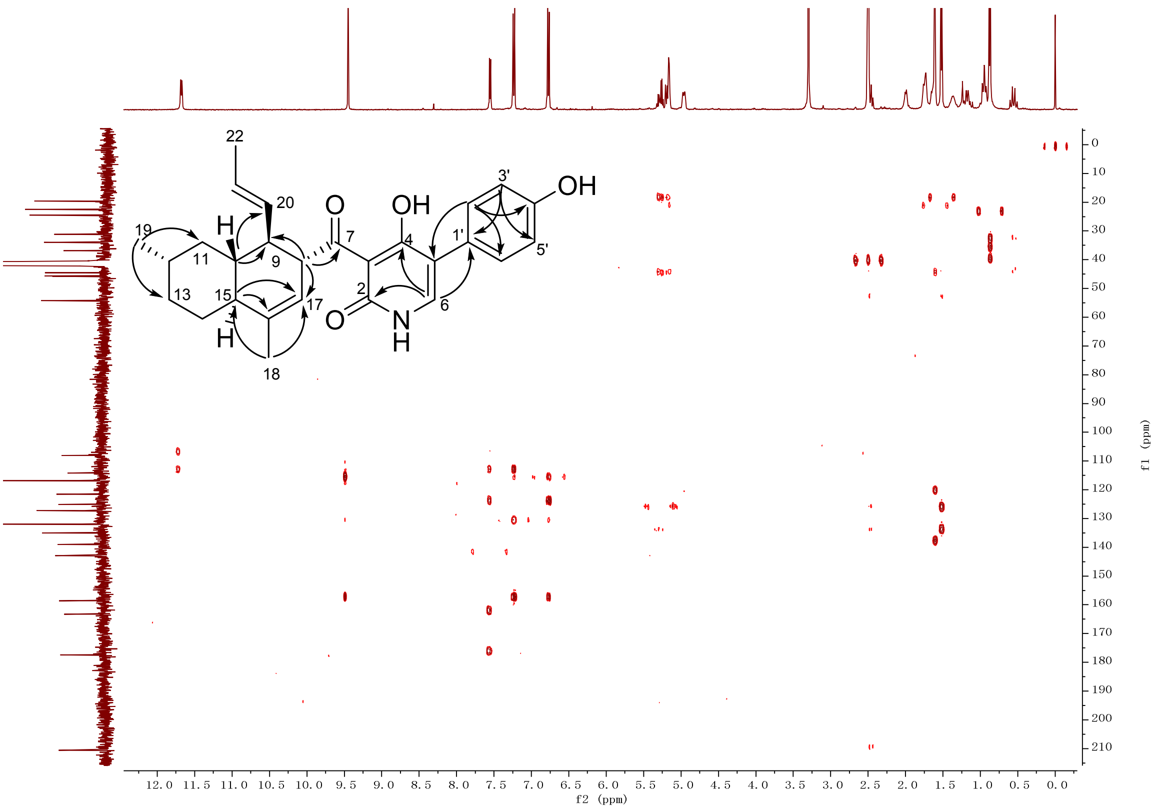

Figure S12. HMBC(⟶) spectrum of ilicicolin H in DMSO-d_6 at 400 MHz

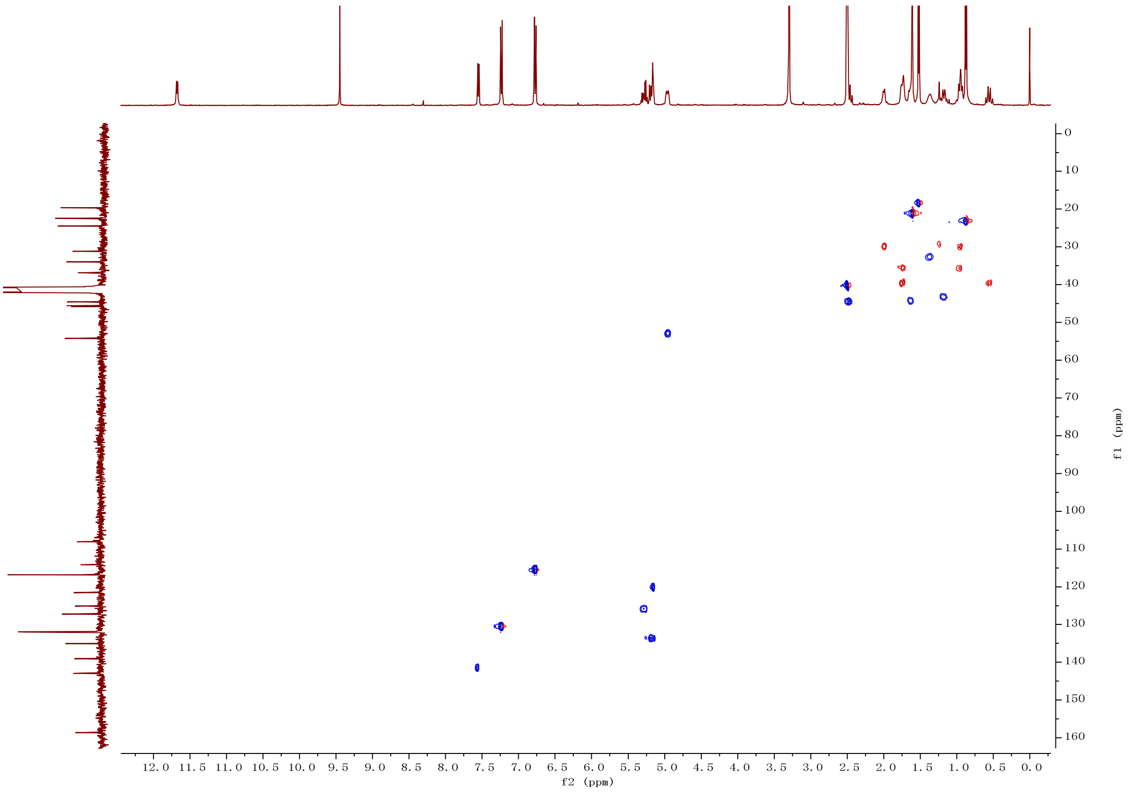

Figure S13. HSQC spectrum of ilicicolin H in DMSO-d_6 at 400 MHz

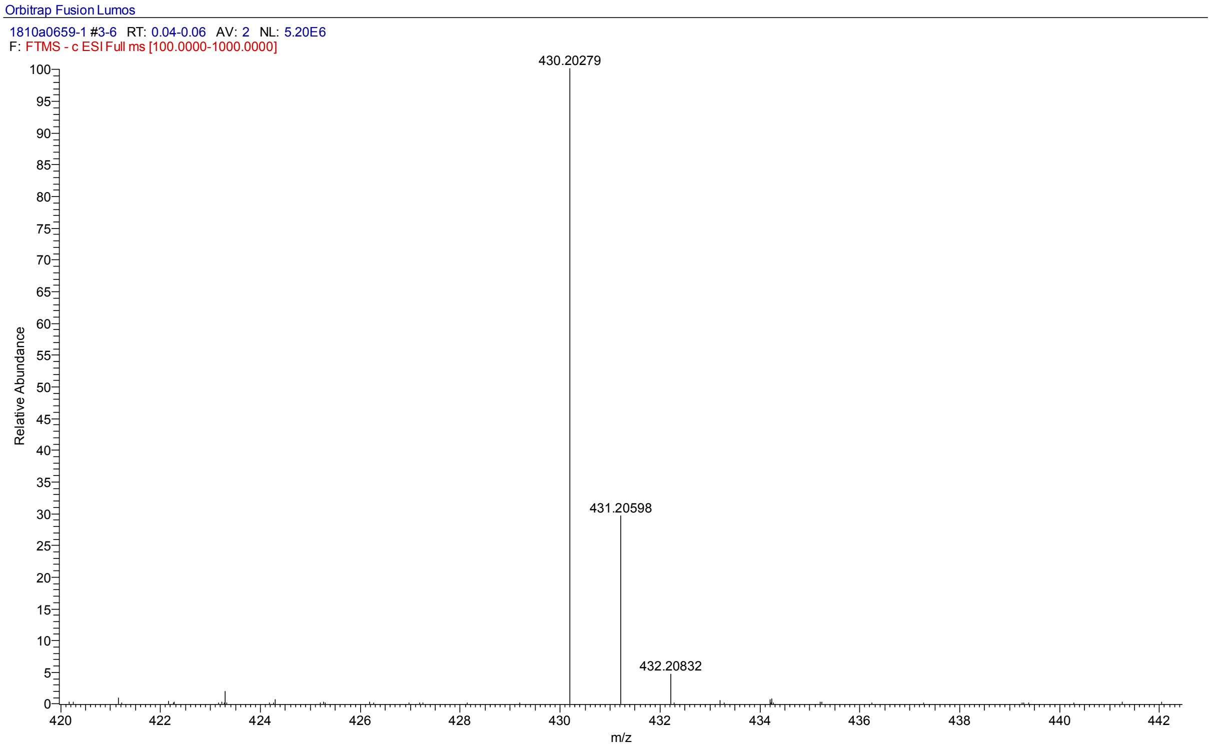

Figure S14. HRMS (ESI, M-H+) of ilicicolin J, calculated for C27H28NO4 430.2024; found 430.2028

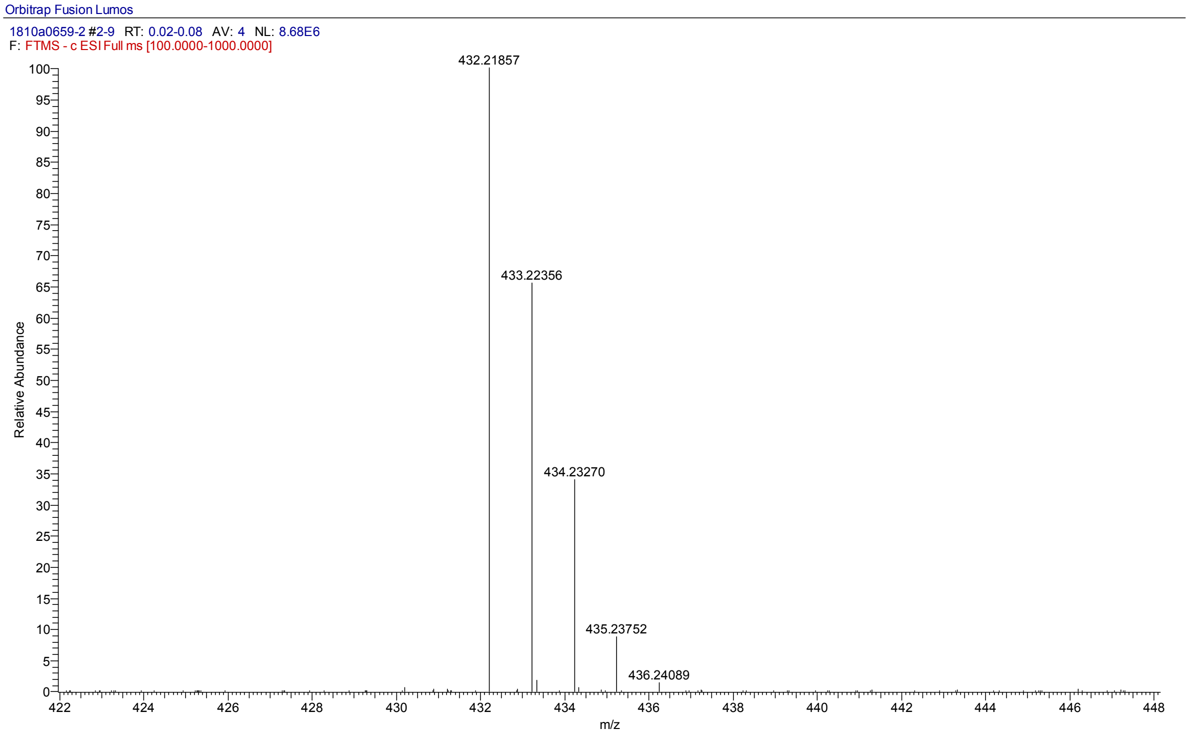

Figure S15. HRMS (ESI, M-H+) of ilicicolin H, calculated for C27H30NO4 432.2180; found 432.2186

SUPPLEMENTARY PROTEIN SEQUENCES:

iliA:4036aa

MSQQRYPEPIAVIGSACRFPGASSSPSKLWSLLQEPRDVLKKFDPDRLNLKRFHHTNGDTH
GATDVNNKSYLLEENTRLFDASFFGISPLEAAGMDPQQRLLLETVYESFEAAGVTLDQLKG
SLTSVHVGVMTNDYSFIQLRDPETLSKYNATGTANSIMSNRISYVFDLKGPSETIDTACSSSLV
ALHHAAQGLLSGDCETAVVAGVNLIFDPSPYITESKLHMLSPDSQSRMWDKSANGYARGE
GAAALLLKPLSRALRDGDHIEGIVRGTGVNSDGQSSGITMPFAPAQSALIRQTYLRAGLDPI
KDRPQYFECHGTGTPAGDPVEARAISESLLDGETSSDNPLYVGSVKTVIGHLEGCAGLAGVI
RAILALKHRTIPPNLHFKELNPAIAQYYGPLQITTKALPWPEVPAGTPARASVNSFGFGGTN
AHAIIESYDNGSASSSIQAQDEQPEESSEGGLGPLIFSAASGSSLLRTVQAYLEHLREHPSVDL
QDLSWLLQTRRTTHRVRTHFSGASRDTVLENMATFVTTHEKASSATIGYQPQLVNPSEAPG
VLGIFTGQGAQWPAMGRELIQKSPLFRKTIEECEAILNALPEGDVPEWSLMQELTADASSSR
LSEAMISQPLCTAVQLGLVNLLTAAGISFDAVVGHSSGEIAATYASGIITTKGAMQIAYYRGF
HAKLATGPDGERGGMLAAGLSFEKATQFCSRPEFQGRIQVAASNAPQSVTLSGDINAIKEA
KEQLDADNIFARQLKVDTAYHSHHMQPCAGPYLQSLLACDIELQAPKPGSCVWNSSVRG
DAELLKRDLSSLKGTYWVANMVQTVLFSQAIESSIWHGGPFDLAIEVGPHPALKGPVEQTL
KAAYGSVPMYTGALKRNGSDVEAFSAALGVTWAQLGPSFVDFSSFREAFYESQAPAPKVIK
DLPTYSWDHEKDYWRESRISRRYRTGKDVGHELLGRRTPDDNDHELRWRNVLKLSEMPW
VRGHEVLDEVLLPGAAYVSIAVEAGKHLATSTGKSVRLIDVENVDILRPVVVPDNQEGVET
LFTAHILSSSPSEGVLRARFSYYICNDQSSGSMVHTCSGDLVVHLGADSESGDLLPPRDAVPP
NLVNIDGERVYKMFEGIDLKYSGVFRSIADSKRCLNYATATGVWPEGSLSNDYGVHPAMLD
VAFQTLFIARAHPASRQITSALLPSHIDRVRVSPSVQILQPEGGGDIKAAFESWVVGQTATSLT
GDLNVYDAETGKTFLQVEGLATNMVGEQDASHDQPIFSKTVWGRYDSVGLADPVRDAVK
DAEATRLAEDIERVALFYIKRIVNQIGADERAGFQWYHQRMFGAFEKHLATIKNDEHPVLP
SNWLADEPSVLEDISNAHPDSIDLQLLHAVGENLADVVRGDTQLLEVMQEDDMLDRFYM
DNCASAPINQSIADVLQQITFKFPRCNILEIGAGTGGTTWSVLNSINNAYDSYTYTDISSGFFP
NAAEKFSDFSNKMAFKILDVEKDPTTQGFVEESYDVIIAANVLHATRSLETTLRNVRSLLKP
GGFLVLMEVTGMQSVRVTFILGGLPGWWLGADDGRPLGPGVSVVDWDVLFDKTGFSGAD
TVMHDLEDDTKHCNSLIVTQAVDDAFLRMREPLSFMAELPPLTEPLLVIGGKKLTTTKMMS
EIQKLLPRSWKRHVQTVGSIDEIDTAKLIPRMDVICLQEADEPLFATPMTAKRIALLKSLLMS
ARNMLWVTGAGKSHTPRTSIFLGIARIVPSELPQLNLQMLGLESGASHSVAARNCVEAFLR
LRATEEGNGSHMLWSQEPEMEILADGQTMVPRVMPNKPLNELYNASRRAVTKTIDATDVP
VRAVAGPGKMTLQAAELQDASAQRARVQVKYALHIPSVNGKQVYLVCGHRQGSESATPV
MAISESNGTIVEVDLERLITIDEDGCTPGVLAATANHLLVRAIATLASGARKVLLYQAEESLA
AMVATEIAAQGGEAHFASSSSDAPDSWIKIHVNSSKRALSRVVPRDVQLYVDCSGYSQSAVS
SVSSASDTLRACVPADCVAQQLGGGLLQEAFQRMDAGGSTLFKDSYAKAKSSFSENQEQIL
DCDLVKAADLAGADASSLTRKRYVTDWQEKESLTLTIQPLDLQGIFKPDKTYFMVGMAGG
LGLSICQWMIRNGAKHLVITSRNPKIDDSLLEDARRANAKLHVMTMDVSKRDSVEKVVRL
VQDTLPPIAGVCNAAMVLSDKLFIDMDVDQLNNTLAAKVYGTEHLDSVFDDMPLDFFVL
LSSVATVIGNIGQANYHAANLFMTSLIAQRRARGLTGSVVHVGYISDVGYVTRQDRDRQLD
QHFRNVRLMPLSETDVHHAFAEAIRGGKPGSVSGAHDIIMGLETFKEPLAPEKQPLWLANP
RFAHFMPPTMLQTQQQHRGSGSADNVRKQVEEAETEDEAVAAVVKAFCSKLESILQLQED
SVNIQRAIIDLGIDSLVAVEIRTWFLKELGAEVAVVKILGGDTVIQVCTWATKKVMAINMKK
KEAAQLDEAAAEKTATAATPAPAPDAAPAPAAPTKTASLTVPVENTSRTPESNSASVSDAD

DSESSGAVSKLGTSISGSSYAKMEFGDADARSESTGSSGMADSDDSSNRPETIREEIMSQAQS
RIWFLSKHLEDPAAFNMTFHYRAQGPLSMARLRHALQVTTHHHECLRMRFYPRLGDGQP
MQGVMGSSLYELEHIPDANDSDVKNELARFKTRVWDLENGKTFGVTVLSHSAEEHDIIYG
YHHLVMDVVGWHVFVHDLDKAYKMQSLDKSAGSYFDYTSLQLEQEKAGVLEEDLKYWQ
AEFTTTPETLPLLPMAHTIVRPAEPGNESHHEYQELTSGQFTALKETCQRLRISPFHFHVAVM
QVLLARYANTEDVCIGIVDANRNDARFAQTVGCFINMLPVRSHVSSHDSFANVARAASKK
ALAAFAHSAVPFDMILDKVKAPRSSASTPLFQAAVNYRTGSVWELPLGDCQMKLAGAKD
ADNPYDISLGITDMGSGCMIEIHCQASLYTSEGCRTILDSYVRLLESFAANPHLDITECEIYDK
SQVGQALELGKGPEMEFGWPSTMSQRVLDMCSLHSDKSAVKDNTITLSYSNLASRVNAVA
DAILQAGCTAGSHVAVLCEPTVDATVAMLAVLHVGAVYVPLDTSLPTARHAAMVQSSRPA
LLLSHSATEGLVRDLGNELDSPIRQVRIDSISEEARQEVPCAAERGAPAVLLFTSGSTGTPKGI
FLSQANFVNHLALKIHVLGFGQECVLQQSSLGFDMSLIQTFCALANGGLLVIVPSEMRRDP
VELTGLLSRERISLTIATPSEYLAWLRYGEASLTENTAWRHACMGGEQVSRQLKSELRRVNLS
GLRLTNCYGPTEITAAATFQAIDLEEKQDEDERAKFAVGKALPNYSVCILDASGQPQPAQH
AGEICIGGAGVALGYLNLADETARKFIADVTSTADRRMYRTGDQGRLLSDGTLLCLGRLDG
DTQVKLRGLRIELQEVESALLQAADGLLSTAVVSQRGDVLVAHATLSPGRDNASEEELTQV
LGHLRLPQYFIPAAIIILAAMPTNSNGKLDRKAIGALSLPERGSNGTQEKMTIREGEVRLLW
ERVLPDTSTTGRLAPSSDFFLCGGNSLLMMKLQAAIRESIGVAISTRTLYQASTLREMARRID
EHQTAEGDDTEREIDWAAETTVPKALLRQIRELPAPVKSSKSDGIEVLMTGATSFLGGHLLQ
ALLRSPVVRKVHCVAVLADDQHQLPRDEKIECYTGSLLSSTLGLNADERDRLEQTVDVIIH
AGSSGHCLNTYDSLRTPNLLSTHFLSSLALPRSIPLLLLSSNRVVLLSGSTAPPPGSVAAFAPA
TDGLEGYTASKWASESFLENLVAHMQQVSRSPLTVAVHRPCVVVSEQAPNSDALNAILRYS
VSMRCVPQLDNVEGYLDFGKVEKIVDEIADSALQLAQAGSQDQEIRFRHHSGGAKVPVRE
FRAHMEDIYGGSFDEVDVTEWMRRAADAGIDPLITAYLEGILDSGSPMVFPYLGEE

iliB:381aa

MALATPLPATQAAVKVTGPSTVDVSAATTLPVLEAFEVLVRVACVSINHVDGKSADMSPTP
GATSGVDFSGLIVALGSKVDSDEFRANNNMRALSIGDRVFGGVFGNNPLRHDNGAFAEYV
AVPARLIWHMPAAMDFSTAATIGATLATVGLALFQYLQVPMPSTQTISDSKTIPDPQQKTR
MALVYGGGTATGAMAIQVLKLAGFRPITTCSPGSAARAMHLGAAATFDYRSPTCGADLRE
HTANGLELALDCITDTASMSICYEALGSAGGRYVALDAFPLRGHTRRSVAAEWVCTYTQFG
HAVAWVPPYNLDARPRDREIAEAWYVVAQQLVDEGLIEPYPKEDRTGGLAAVGEGMRAV
WKGEISGRKLAYPIAEECY

iliC:505aa

MITNDLIAQHSLTLTIASSVLLVFLLSRLLRKDATGKAQGCRPVAKRWQWDPILGLDIVLAQ
IGALKGNYYLPWLIELHSNMPKTFEINFFGKRQIYTSEPDNLKAMTATNFHDFGIEPMRRHT
KGSMPFADKGISTVDGKEWEFSRFLLKPFFYREVYTSTDRIEPFADHMMALIPGDGESFNMQ
SLIQRWFLDLTTNFIFGKPMDALENPDRARITWAMLDVLKGGRLRAQFYMMMWAFNWT
WWYKAVAEVHDFINVHIRETYKEIEEREQRIKDGKPVEPERTDLIWYMAWNLRDEELLRSQ
LCLVFVPNNDTTSIFISNCIWHLARHPEAWEKLRQEVLAHGDAPLTFEALRNMKYLQCVL
NETHRLTPNNVTQIRVCLNDSVLPVGGGKNAKEPFFVRKGDVVSITKTVMYRDPEIWGND
AEEFKPERFDGRRVFWEFLPFGGGPRRCPAQMMVQTEAAYMLARLARVYRRIEARDPAPY
TAVMRIGPSNKTGVQIAVYK

iliD:244aa

MTSTEAAGTGKAPAIRANPALQTYYESQESYLVYEVVLRGSHHFGFYEKDTYWPFPVGRSL
ERMEAKLLSALALPSGSQILDAGCGFGPVAISMAKKGMRVTAIDIIDHHVTKARRNVEKAG
LPKGQVTVEKMDYQHLESIASESHDDAKAAATGFFRILKPGGRIAFFEAQRSRTSGDYDEG
DELAGHLKLVNEYTAMPTNELSREDYFKDLLEDAGFVDVEFTLPPGTREPREHWSYSALKA

iliE:766aa

MSEQLGSHITTPSSHDDASKDKRPAAEVVNGSGIFIMADLHTGKPITLKCGLTLPNRLVKAA
TAESMAPNNTLPDEKFQNLYRHWAEGGWGMVLAGNVQVDANHICTATDLSVDHSLSDS
KIVEAWRPWAAACNGNGTVTVMQLCHPGRQSPAGAGKRGLFAKSIAPSAVALQMGSGLV
AKAVTALLFGTPREMSVSDIETVVSQFARSARLAAESGFAGVEVHAGHGFLLEQFLSTKSNR
RTDAYGGTPAKRARIVVEVLTAIRAVVPAGFCVGLSLNSVDLQSQTELKDCVEQVKLITDAG
VDFIEVSGGTFENPTMFLGPEKSRKQAQLGQPLAHEPFFLDFAKAIRPHVPGVPLIVTGGFR
SCQGIEETIAGGDADLVGLARPAVVNPLLPKTTVLSPKTTEFGPEIEDGDVTLYAKKTEAPWI
LKQIGIRAVEVHIDNSVYHNRRHAAKQVRRASVLLQFPSRPSLSVAAVDIDNVISRLSTARPF
VFIFLAITVEVNIDTDIAALLLALRPSPELYHLAAAMPPRRSDGSADHDVPDWPKTPHPTPY
DILAMRKDDPYTKHRFFQLVKIYHPDRHGHTPAVHRLPHATRLERYRLIVAANDLLSNPSK
RSLYDTQGVGWTGDRPPTLNESVRHAEKSWRHQPGNASRNATWEDWERWYDARDGKT
RDPMYMSNGVFATLVVMMCMIGAFAQMSRAEQSGTEYLETRDQSNLAIGQQISRTTLVSA
GRSKDERVDSFLRERENVAYEFTPSKYDDRTRTEA
